# *Ramlibacter terrae* sp. nov. and *Ramlibacter montanisoli* sp. nov., Isolated from Soil

**DOI:** 10.4014/jmb.2105.05023

**Published:** 2021-07-21

**Authors:** Shehzad Abid Khan, Hyung Min Kim, Ju Hye Baek, Hye Su Jung, Che Ok Jeon

**Affiliations:** Department of Life Science, Chung-Ang University, Seoul 06974, Republic of Korea

**Keywords:** *Ramlibacter terrae*, *Ramlibacter montanisoli*, *Betaproteobacteria*, new taxa, soil

## Abstract

Two gram-negative, catalase-positive, strictly aerobic, and white colony-forming bacteria, strains H242^T^ and B156^T^, were isolated from soil in South Korea. Cells of strain H242^T^ were oxidase-positive and non-motile short rods, while those of strain B156^T^ were oxidase-negative and long non-motile rods. Ubiquinone-8 was identified as the sole isoprenoid quinone in both strains. C_16:0_, cyclo-C_17:0_, and summed feature 3 (C_16:1_ *ω7c* and/or C_16:1_ *ω6c*) and phosphatidylethanolamine, phosphatidylglycerol, and diphosphatidylglycerol were identified in both strains as the major cellular fatty acids and polar lipids, respectively. The DNA G+C contents of strains H242^T^ and B156^T^ were 69.4 mol% and 69.3 mol%, respectively. Phylogenetic analyses based on 16S rRNA and 92 concatenated core gene sequences revealed that strains H242^T^ and B156^T^ formed distinct phylogenic lineages from other *Ramlibacter* type strains. The DNA-DNA hybridization (DDH) value between strains H242^T^ and B156^T^ was 24.6%. Strains H242^T^ and B156^T^ were most closely related to *Ramlibacter ginsenosidimutans* BXN5-27^T^ and *Ramlibacter monticola* G-3-2^T^ with 98.4% and 98.6% 16S rRNA gene sequence similarities, respectively. Digital DDH values between strain H242^T^ and *R. ginsenosidimutans* and between strain B156^T^ and *R. monticola* were 23.5% and 26.1%, respectively. Phenotypic, chemotaxonomic, and molecular analyses indicated that strains H242^T^ and B156^T^ represent two novel species of the genus *Ramlibacter*, for which the names *Ramlibacter terrae* sp. nov. and *Ramlibacter montanisoli* sp. nov., respectively, are proposed. The type strains of *R. terrae* and *R. montanisoli* are H242^T^ (=KACC 21667^T^ =JCM 33922^T^) and B156^T^ (=KACC 21665^T^ =JCM 33920^T^), respectively.

## Introduction

The genus *Ramlibacter*, a new genus of the family *Comamonadaceae* in the class *Betaproteobacteria*, was first proposed by Heulin *et al*. with *Ramlibacter tataouinensis* as the type species, which was isolated from subdesert soil [1]. At the time of writing, the genus comprises 10 validly published species (https://lpsn.dsmz.de/genus/ramlibacter) isolated primarily from soil habitats, such as subdesert soil [1], ginseng soil [2, 3], forest soil [4-6], and garden soil [7, 8]. An exception is *Ramlibacter aquaticus* LMG 30558T, which was isolated from a water environment [9]. The genus *Ramlibacter* includes gram-negative, catalase-positive, oxidase-variable, aerobic, and non-motile (by flagella) rod or coccoid bacteria containing ubiquinone-8 (Q-8) and C_16:0_, cyclo-C_17:0_, and summed feature 3 (C_16:1_ *ω7c* and/or C_16:1_ *ω6c*) as the major respiratory quinone and fatty acids, respectively [1-9]. The DNA G+C content of *Ramlibacter* species ranges from 62.0–70.6 mol%. In this study, we isolated two strains, presumably representing novel species of the genus *Ramlibacter*, from soil samples collected from different areas in South Korea and taxonomically characterized them using a polyphasic approach.

## Material and Methods

### Isolation and Cultivation

Strains H242^T^ and B156^T^ were isolated from flatland near a river (37°15′04.2′′N, 128°32′26.3′′E) and mountain (37°15′38.7′′N, 128°36′28.6′′E), respectively, in Gangwon Province of South Korea, according to a previously described procedure [10]. In brief, soil samples were collected in sterile plastic tubes and stored in an icebox for transport. The soil samples were resuspended and serially diluted in phosphate-buffered saline (137 mM NaCl, 2.7 mM KCl, 10 mM Na_2_HPO_4_, 2 mM KH_2_PO_4_, pH 7.2). The serially diluted aliquots (100 μl) were spread on R2A agar (BD, USA), and the agar plates were incubated aerobically at 30 °C for 3 d. The 16S rRNA genes of colonies grown on R2A agar were PCR-amplified using the universal primers F1 (5′-AGA GTT TGA TCM TGG CTC AG-3′) and R13 (5′-TAC GGY TAC CTT GTT ACG ACT T-3′) and double-digested with HaeIII and HhaI. The representative PCR amplicons of 16S rRNA genes showing different fragment patterns were partially sequenced using the universal primer 340F (5′-CCT ACG GGA GGC AGC AG-3′) at Macrogen (Korea). The resulting sequences were compared with all validly reported type strains using the Nucleotide Similarity Search program on the EzBi°Cloud server (http://www.ezbiocloud.net/identify) [11]. From the comparative analysis, strains H242^T^ and B156^T^ were selected as putative novel species of the genus *Ramlibacter* for further analyses. For full 16S rRNA gene sequencing, the PCR amplicons of 16S rRNA genes of strains H242^T^ and B156^T^ were further sequenced using universal primers 518R (5′-ATT ACC GCG GCT GCT GG-3′) and 805F (5′-GAT TAG ATA CCC TGG TAG TC-3′), and the nucleotide sequences obtained using the 340F, 518R, and 805F primers were assembled. Strains H242^T^ and B156^T^ were routinely cultured on R2A agar and/or in R2A broth for 2 d at 30°C and stored at –80°C in R2A broth containing 15% (v/v) glycerol for long-term preservation. *R. tataouinensis* KACC 11924T, *Ramlibacter ginsenosidimutans* KACC 17527T, *Ramlibacter monticola* KACC 19175T, and *Ramlibacter alkalitolerans* KACC 19305^T^ were obtained from the Korean Agricultural Culture Collection Center and used as reference strains for the comparison of phenotypic properties and fatty acid composition.

### Phylogenetic Analysis Based on 16S rRNA Gene Sequences

The 16S rRNA gene sequence similarities of strains H242^T^ and B156^T^ with other bacterial type strains were calculated using the EzBi°Cloud server. The 16S rRNA gene sequences of strains H242^T^ and B156^T^ and closely related type strains were aligned using the fast secondary-structure-aware infernal aligner available in the Ribosomal Database Project (RDP) [12]. Phylogenetic trees using the neighbor-joining (NJ), maximum-likelihood (ML), and maximum-parsimony (MP) algorithms with bootstrap values (1,000 replications) were constructed using MEGA7 software [13] based on the Kimura two-parameter model, the nearest-neighbor-interchange heuristic search method, and the complete deletion options, respectively. Taxonomic classifications of strains H242^T^ and B156^T^ were confirmed using the RDP Naïve Bayesian rRNA Classifier (ver. 2.11; http://rdp.cme.msu.edu/classifier/) [12] based on RDP 16S rRNA training set 16.

### Genome Sequencing and Phylogenomic Analysis

For whole genome sequencing, the genomic DNA of strains H242^T^ and B156^T^ was extracted using the phenol-chloroform extraction and ethanol precipitation method [14] and sequenced with an Oxford Nanopore MinION sequencer (Nanopore, UK). The resulting sequencing reads were *de novo* assembled using Unicycler (version 0.4.7) [15], and the quality of the assembled genomes was checked on the basis of their completeness and contamination rates using the CheckM program (version 1.0.4) [16]. Average nucleotide identity (ANI) and digital DNA-DNA hybridization (DDH) values among the genomes of strains H242^T^ and B156^T^ and closely related type strains were calculated using the stand-alone software available on the EzBi°Cloud server (www.ezbiocloud.net/sw/oat) [17] and the server-based Genome-to-Genome Distance Calculator version 2.1 http://ggdc.dsmz.de/distcalc2.php) [18], respectively. House-keeping core genes were extracted from the genome sequences of strains H242^T^ and B156^T^ and closely associated type strains using the up-to-date bacterial core gene (UBCG) pipeline (www.ezbiocloud.net/tools/ubcg) [19]. A phylogenomic ML tree with bootstrap values (1,000 replications) based on 92 concatenated housekeeping core genes was constructed using MEGA7 software.

### Phenotypic, Physiological, and Biochemical Analyses

The growth of strains H242^T^ and B156^T^ was tested on R2A agar, tryptic soy agar (TSA; BD), nutrient agar (NA; BD), marine agar (MA; BD), and laboratory-prepared Luria– Bertani agar for 3 d at 30°C. The growth of strains H242^T^ and B156^T^ was evaluated at different temperatures (4, 10, 15, 20, 25, 30, 37, 40, and 45°C) and pH values (5.0–11.0 at 0.5 pH unit intervals) using R2A agar and R2A broth, respectively, for 3 d. R2A broth with pH 5.0–5.5, pH 6.0–7.5, and pH 8.0–11.0 was prepared using sodium citrate, Na_2_HPO_4_/NaH_2_PO_4_, and Tris-HCl buffers, respectively [20], and their pH values were adjusted if necessary after autoclaving (at 121°C for 15 min). The growth of strains H242^T^ and B156^T^ was examined in R2A broth with different NaCl concentrations (0%–4% at 0.5% intervals, w/v). The biochemical and physiological properties of strains H242^T^ and B156^T^ were tested using cells grown on R2A agar after aerobic incubation for 2 d at 30°C. Gram staining was performed using a Gram stain kit (bioMérieux, France), according to the manufacturer’s instructions. The gliding motility of strains H242^T^ and B156^T^ was tested using R2A containing 0.3% agar as described previously [21]. The cell morphology of strains H242^T^ and B156^T^ was inspected using phase-contrast microscopy (Carl Zeiss, Germany) and transmission electron microscopy (JEM–1010; Jeol, Japan). Oxidase and catalase activities were tested by oxidation of 1% (w/v) tetramethyl-p-phenylenediamine (Merck, USA) and by the production of oxygen bubbles in 3% (v/v) aqueous hydrogen peroxide solution, respectively [22]. To assess the anaerobic growth of strains H242^T^ and B156^T^, both strains were streaked on R2A agar and incubated at 30°C for 21 days under anaerobic conditions prepared using the GasPak Plus system (BBL, USA). The properties of strains H242^T^ and B156^T^ and their four closely related reference strains were investigated in parallel under the same growth conditions. Hydrolysis of tyrosine, casein, esculin, gelatin, starch, Tween 20, and Tween 80 was tested on R2A agar following the methods described by Lányi and Tindall *et al*. [22, 23]. Additional biochemical features and enzymatic activities were tested using the API 20NE and API ZYM systems (bioMérieux), respectively, according to the manufacturer’s instructions.

### Chemotaxonomic Analyses

For the analysis of respiratory quinones, strains H242^T^ and B156^T^ were aerobically cultivated to their exponential growth phases in R2A broth at 30°C. Microbial cells were harvested and their isoprenoid quinones were extracted, according to the method of Minnikin *et al*. [24], and analyzed using a model LC-20A HPLC system (Shimadzu, Japan) equipped with a diode array detector (SPD-M20A; Shimadzu) and a reversed-phase column (250×4.6 mm, Kromasil; Akzo Nobel, Japan). For cellular fatty acid analysis, strains H242^T^ and B156^T^ and four reference strains were cultivated in R2A broth at their optimal temperatures and their microbial cells were harvested at the same growth stage (exponential phase, optical density, OD_600_ = 0.8). Cellular fatty acids of microbial cells were saponified, methylated, and extracted using the standard MIDI protocol. Fatty acid methyl esters were analyzed by gas chromatography (Hewlett Packard 6890, USA) and identified using the RTSBA6 database of the Microbial Identification System (Sherlock ver. 6.0B) [25]. Polar lipids of strains H242^T^ and B156^T^ were extracted from cells harvested during the exponential growth phase and analyzed by two-dimensional thin-layer chromatography (TLC), according to the procedure described by Minnikin *et al*. [26]. The following reagents were used to identify different polar lipids: 10% ethanolic molybdophosphoric acid (for total polar lipids), ninhydrin (for aminolipids), Dittmer-Lester reagent (for phospholipids), and *α*-naphthol/sulfuric acid (for glycolipids). Major polar lipids (diphosphatidylglycerol, phosphatidylglycerol, and phosphatidylethanolamine) identified from strains H242^T^ and B156^T^ were confirmed using standard polar lipid compounds purchased from Sigma-Aldrich (USA).

## Results and Discussion

### Molecular Phylogenetic Analysis

**Fig. 1:**
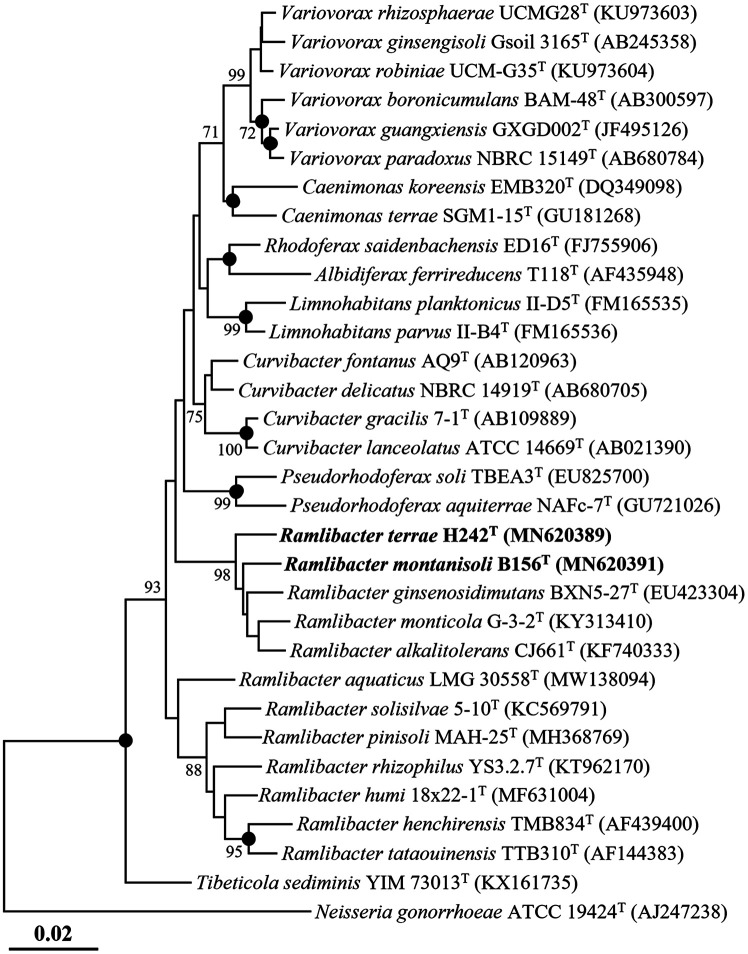
A neighbor-joining tree (NJ) showing the phylogenetic relationships between strains H242^T^ and B156^T^ and closely related species, based on 16S rRNA gene sequences. Bootstrap values (based on 1,000 replication) greater than 70% are shown at branch points. The filled circles (●) indicate branches that were commonly recovered using the NJ, maximum likelihood, and maximum parsimony algorithms. *Neisseria gonorrhoeae* ATCC 19424^T^ (AJ247238) was used as an outgroup. Scale bar, 0.02 substitutions per nucleotide position.

Almost complete 16S rRNA gene sequences of strains H242^T^ (1,460 nucleotides) and B156^T^ (1,453 nucleotides) were obtained through sequencing and assembly of their 16S rRNA gene amplicons using the 340F, 518R, and 805F primers. Comparative analysis using the EzTaxon program based on the 16S rRNA gene sequences revealed that strain H242^T^ was most closely related to *R. ginsenosidimutans* BXN5-27^T^ and *R. monticola* G-3-2^T^ with 98.4%and 97.7% 16S rRNA gene sequence similarities, respectively, whereas strain B156^T^ was most closely related to *R. monticola* G-3-2^T^ and *R. alkalitolerans* CJ661^T^ with 98.7% and 98.3% 16S rRNA gene sequence similarities, respectively. Phylogenetic analysis based on 16S rRNA gene sequences using the NJ algorithm revealed that strains H242^T^ and B156^T^ formed distinct phylogenic lineages from other *Ramlibacter* type strains (Fig. 1), as did the ML and MP algorithms (Fig. S1). Taxonomic analysis of strains H242^T^ and B156^T^ using the RDP classifier tool also suggested that strains H242^T^ and B156^T^ could be classified as unclassified species of the genus *Ramlibacter*. Sequence similarity and phylogenetic analyses based on 16S rRNA gene sequences suggested that strains H242^T^ and B156^T^ could represent novel species of the genus *Ramlibacter*.

### Genomic and Phylogenomic Analyses

**Fig. 2:**
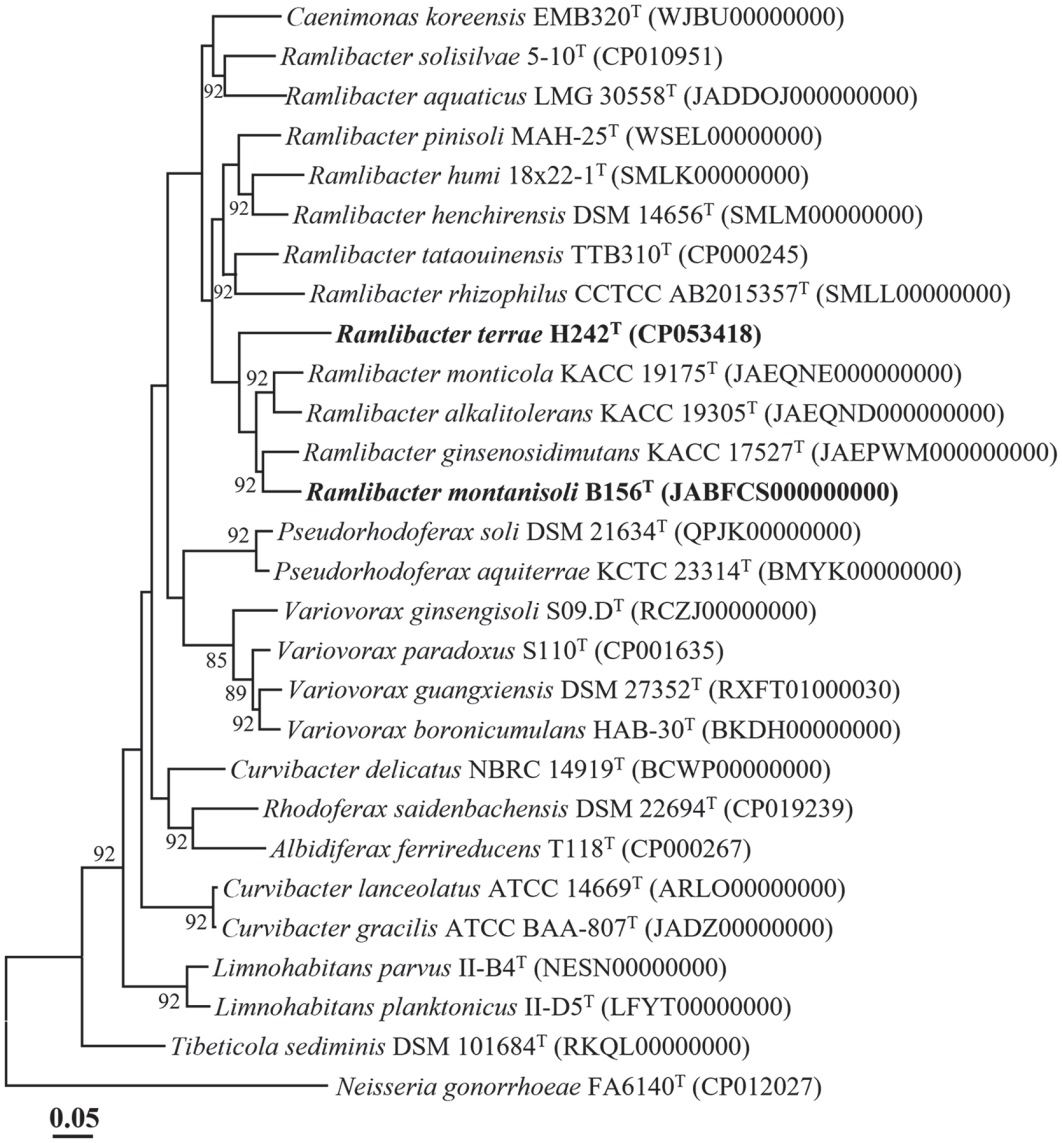
A phylogenomic tree showing the phylogenetic relationships between strains H242^T^ and B156^T^ and closely related species, based on the 92 concatenated housekeeping core gene sequences. Bootstrap values (based on 1,000 replication) greater than 70% are shown at branch points. *Neisseria gonorrhoeae* FA6140^T^ (CP012027) was used as an outgroup. Bar, 0.05 substitutions per nucleotide position.

The *de novo* assembly of the genome sequencing data of strain H242^T^ resulted in a complete genome with an average genome coverage of 306.0×, whereas that of strain B156^T^ produced two contigs that were 4,459,501 bp and 293,893 bp in size, with an average genome coverage of 246.0×. The completeness values of strains H242^T^ and B156^T^ were 95.6% and 93.4%, respectively, and the contamination rates of these strains were 0% and 1.6%, respectively, and all values met the criteria for high-quality genomes (completeness ≥ 90% and contamination ≤ 10%) [16]. The complete genome of strain H242^T^ was 5,277,406 bp in size, and 5,653 total genes, 2,807 protein-coding genes, and 45 tRNA genes encoding 20 amino acids were predicted from the genome. The draft genome of strain B156^T^ was 4,753,394 bp in size, and 4,741 total genes, 3,639 protein-coding genes, and 44 tRNA genes encoding 20 amino acids were predicted from the genome. The 16S rRNA gene sequences of strains H242^T^ and B156^T^ obtained using the PCR approach were identical to those in their genomes. The G+C contents of strains H242^T^ and B156^T^ calculated from their genomes were 69.4 mol% and 69.3 mol%, respectively, which were in the ranges of G+C contents of *Ramlibacter* species. The ANI and digital DDH values between strains H242^T^ and B156^T^ were 81.9% and 24.6%, respectively, suggesting that strains H242^T^ and B156^T^ are different species of the genus *Ramlibacter*. The ANI and digital DDH values between strain H242^T^ and *R. ginsenosidimutans* KACC 17527T, the most closely related type strain to strain H242^T^, were 80.2% and 23.5%, respectively. Those between strain B156^T^ and *R. monticola* KACC 19175T, the most closely related type strain to strain B156^T^, were 83.1% and 26.1%, respectively. These values were clearly lower than the thresholds (ANI, ~95%; digital DDH, 70%) for prokaryotic species delineation [27]. Additionally, the phylogenomic tree based on 92 orthologous housekeeping core genes revealed that strains H242^T^ and B156^T^ formed distinct phylogenetic lineages within the genus *Ramlibacter* (Fig. 2). In conclusion, the genome relatedness and phylogenomic analyses of strains H242^T^ and B156^T^ with closely related type strains clearly suggested that they could represent two novel species of the genus *Ramlibacter*.

### Phenotypic, Physiological, and Biochemical Characteristics

Both strains H242^T^ and B156^T^ grew well on R2A agar and exhibited relatively good growth on NA and TSA, but did not grow on MA. Strain H242^T^ grew slowly on LB agar, whereas strain B156^T^ did not grow on it. Both strains formed white colonies on R2A agar. Cells of strains H242^T^ were gram-negative and non-motile short rods, 0.6–0.7 μm in width, and 1–1.2 μm in length, whereas those of strain B156^T^ were gram-negative and non-motile long and slender rods, 0.3–0.4 μm in width, and 1.8–2.2 μm in length (Fig. S2). Neither strain showed any growth under anaerobic conditions, suggesting that they are strictly aerobic. Many phenotypic properties, such as reduction of nitrate, activity of catalase, esterase lipase (C8), leucine arylamidase, and naphthol-AS-BI-phosphohydrolase, and assimilation of D-glucose, D-mannose, D-mannitol, and malic acid, were common between strains H242^T^ and B156^T^ and their closely related *Ramlibacter* species, but many other phenotypic properties, described in Table 1, differentiated strains H242^T^ and B156^T^ from their closely related *Ramlibacter* species.

### Chemotaxonomic Characteristics

Q-8 was detected as the sole respiratory quinone from strains H242^T^ and B156^T^, which is in agreement with other species of the genus *Ramlibacter* [1-9]. Of the major cellular fatty acids (> 10% of the total fatty acids), strain H242^T^ contained C_16:0_ (26.9%), cyclo-C_17:0_ (16.5%), summed feature 3 (C_16:1_ *ω7c* and/or C_16:1_ *ω6c*, 20.3%) and summed feature 8 (C_18:1_ *ω7c* and/or C_18:1_ *ω6c*, 13.2%), and strain B156^T^ contained C_16:0_ (28.4%), cyclo-C_17:0_ (10.7%), and summed feature 3 (C_16:1_ *ω7c* and/or C_16:1_ *ω6c*, 22.4%). The overall fatty acid profiles of strains H242^T^ and B156^T^ were generally similar to those of closely related *Ramlibacter* reference strains, but there were some differences in both components and compositions (Table 2). For example, C_10:0_ was not detected in strain H242^T^ but was detected in other strains, and iso-C_16:0_ was not detected in strain B156^T^ but was detected in *R. monticola*, the most closely related species. C_10:0_ 3-OH was detected in strains H242^T^ and B156^T^ as the sole hydroxy fatty acid. Phosphatidylglycerol, diphosphatidylglycerol, and phosphatidylethanolamine were identified from strains H242^T^ and B156^T^ as the major polar lipids, and an unidentified aminophospholipid was additionally identified from strain B156^T^ as a minor polar lipid (Fig. S3). The polar lipid profiles of both strains were in accordance with those of other *Ramlibacter* species [2, 5]. In conclusion, the phylogenetic analysis, genome relatedness, and phenotypic and chemotaxonomic features suggest that strains H242^T^ and B156^T^ represent two novel species of the genus *Ramlibacter*, for which the names *Ramlibacter terrae* sp. nov. and *Ramlibacter montanisoli* sp. nov. are proposed.

### Description of *Ramlibacter terrae* sp. nov.

*Ramlibacter terrae* (ter'rae. L. gen. n. terrae of the soil).

Cells are gram-negative, strictly aerobic, and short rods without flagellum (0.6–0.7 μm in width and 1–1.2 μm in length). Gliding motility is negative. Colonies on R2A agar are white-colored, circular, smooth, and convex being 1–2 mm in diameter after 3 d of incubation at 30°C. Growth occurs at 10–30°C (optimum, 30°C), pH 6.0–9.0 (optimum, 7.0–7.5), and in the presence of 0–4.5% NaCl (optimum, in R2A broth without the addition of NaCl). Catalase- and oxidase-positive. Nitrate is reduced to nitrite and nitrogen gas is produced. Indole production and glucose fermentation are negative. Hydrolyzes gelatin and esculin, but not Tween 80, urea, casein, Tween 20, tyrosine, and starch. Positive for alkaline phosphatase, esterase (C4), lipase (C14), esterase lipase (C8), valine arylamidase, acid phosphatase, leucine arylamidase, cystine arylamidase, and naphthol-AS-BI-phosphohydrolase activity, but negative for arginine dihydrolase, trypsin, *α*-chymotrypsin, *α*-galactosidase, *β*-galactosidase, *β*-glucuronidase, *α*-glucosidase, *β*-glucosidase, *N*-acetyl-*β*-glucosaminidase, *α*-mannosidase, and *α*-fucosidase activity. Assimilation of D-glucose, D-mannose, D-mannitol, malic acid, L-arabinose, *N*-acetyl-glucosamine, and trisodium citrate is positive, but assimilation of D-maltose, potassium gluconate, capric acid, adipic acid, and phenyl acetate is negative. Q-8 is the sole isoprenoid quinone. The major cellular fatty acids (> 10%) are C_16:0_, cyclo-C_17:0_, summed feature 3 (C_16:1_ *ω7c* and/or C_16:1_ *ω6c*), and summed feature 8 (C_18:1_ *ω7c* and/or C_18:1_ *ω6c*). Phosphatidylglycerol, diphosphatidylglycerol, and phosphatidylethanolamine are the major polar lipids.

The type strain is H242^T^ (=KACC 21667^T^ =JCM 33922^T^), isolated from flatland soil of Gangwon Province in South Korea. The DNA G+C content calculated from the whole genome sequence of the type strain is 69.4 mol%. The GenBank accession numbers of the 16S rRNA gene and genome sequences of strain H242^T^ are MN620389 and CP053418, respectively.

### Description of *Ramlibacter montanisoli* sp. nov.

*Ramlibacter montanisoli* (mon.ta.ni.sóli. L. masc. adj. *montanus* of a mountain; L. neut. n. *solum* soil; N.L. gen. n. *montanisoli* of mountain soil).

Cells are gram-negative, strictly aerobic, and long and slender rods without flagella (0.3–0.4 μm in width and 1.8–2.2 μm in length). Gliding motility is negative. Colonies on R2A agar are white-colored, circular, smooth, and convex being 1–1.5 mm in diameter after 3 d of incubation at 30°C. Catalase-positive and oxidase-negative. Growth occurs at 20–40°C (optimum, 30°C) and pH 6.0–9.0 (optimum, 7.0–7.5) and in R2A broth (does not grow in R2A broth with the addition of 0.5% NaCl). Nitrate is reduced to nitrite and nitrogen gas is produced. Indole production and glucose fermentation are negative. Hydrolyzes esculin and gelatin, but not casein, urea, Tween 80, Tween 20, tyrosine, and starch. Positive for alkaline phosphatase, esterase (C4), lipase (C14), esterase lipase (C8), leucine arylamidase, valine arylamidase, cystine arylamidase, acid phosphatase, and naphthol-AS-BI-phosphohydrolase activity, but negative for trypsin, *α*-chymotrypsin, *α*-galactosidase, *β*-galactosidase, *α*-glucosidase, *β*-glucosidase, *β*-glucuronidase, *N*-acetyl-*β*-glucosaminidase, *α*-mannosidase, and *α*-fucosidase activity. Assimilation of D-glucose, D-mannose, D-mannitol, malic acid, and D-maltose is positive, but assimilation of L-arabinose, capric acid, potassium gluconate, *N*-acetyl-glucosamine, adipic acid, trisodium citrate, and phenyl acetate is negative. Q-8 is the sole isoprenoid quinone. The major cellular fatty acids (> 10%) are C_16:0_, cyclo-C_17:0_, and summed feature 3 (C_16:1_ *ω7c* and/or C_16:1_ *ω6c*). Phosphatidylglycerol, diphosphatidylglycerol, and phosphatidylethanolamine are the major polar lipids and a minor polar lipid, an unidentified aminophospholipid, is also present.

The type strain is B156^T^ (=KACC 21665^T^ =JCM 33920^T^), isolated from mountain soil of Gangwon province in South Korea. The DNA G+C content calculated from the whole genome sequence of the type strain is 69.3 mol%. The GenBank accession numbers of the 16S rRNA gene and genome sequences of strain B156^T^ are MN620391 and JABFCS000000000, respectively.

## Supplemental Materials

Supplementary data for this paper are available on-line only at http://jmb.or.kr.

## Figures and Tables

**Table 1:** Differential phenotype characteristics of strains H242^T^ and B156^T^ and closely related type strains of the genus *Ramlibacter*.

Characteristic	1*	2*	3	4	5	6
Isolation source	Forest soil	Forest soil	Forest soil	Ginseng field	Ginseng field	Desert soil
Colony color	White	White	Brown	White	White	Yellow-orange
Cell morphology	Short rods	Long rods	Short rods	Long rods	Short rods	Short rods
Oxidase	+	–	–	+	+	+
Gliding motility	–	–	–	+	–	+
Optimum growth at:						
Temperature (°C)	30	30	20–32	25–37	30	30
pH	7.0–7.5	7.0–7.5	6.5–8.0	7.0	7.0	7.5
Hydrolysis* of:						
Casein, starch	–	–	–	+	–	+
Tween 80, Tween 20, tyrosine	–	–	–	+	–	–
Enzyme activity (API ZYM)* of:						
Alkaline phosphatase, lipase (C14)	+	+	–	+	+	–
Esterase (C4)	+	+	–	+	+	+
Valine arylamidase, acid phosphatase	+	+	+	+	+	–
Cystine arylamidase	+	+	–	–	–	–
*β*-Glucosidase, *N*-acetyl-*β*-glucosaminidase	–	–	–	+	–	–
Gelatinase	+	–	+	–	+	–
Urease	–	–	+	–	–	–
Assimilation (API 20NE)* of:						
L-Arabinose	+	–	+	+	–	–
*N*-Acetyl-glucosamine	+	–	+	–	–	+
D-Maltose	–	+	+	–	+	+
Potassium gluconate	–	–	+	–	–	+
Trisodium citrate	+	–	+	–	+	–
DNA G+C content (mol%)^†^	69.4	69.3	69.3	68.7	69.2	70.0

Taxa: 1, strain H242^T^ (this study); 2, strain B156^T^ (this study); 3, *R. monticola* KACC 19175^T^ [5]; 4, *R. ginsenosidimutans* KACC 17527^T^ [3]; 5, *R. alkalitolerans* KACC 19305^T^ [2]; 6, *R. tataouinensis* KACC 11924^T^ [1]. All strains are aerobic and positive for the following characteristics: catalase activity, nitrate reduction*, esculin hydrolysis*, activity* of esterase lipase (C8), leucine arylamidase, and naphthol-AS-BI-phosphohydrolase, and assimilation* of D-glucose, D-mannose, D-mannitol, and malic acid. All strains are negative for the following characteristics: flagellar motility, indole production, glucose fermentation, activity* of arginine dihydrolase, trypsin, *α*-chymotrypsin, *α*-galactosidase, *β*-galactosidase, *β*-glucuronidase, *α*-glucosidase, *α*-mannosidase, and *α*-fucosidase, and assimilation* of capric acid, adipic acid, and phenyl acetate. Symbols: +, positive; –, negative.

*These analyses were conducted under the same conditions used in this study.

^†^The DNA G+C contents were calculated from the whole genome sequences in this study.

**Table 2:** Cellular fatty acid compositions (%) of strains H242^T^ and B156^T^ and closely related type strains of the genus *Ramlibacter*.

Fatty acid	1	2	3	4	5	6
Saturated:						
C_10:0_	–	3.2	tr	tr	tr	2.1
C_12:0_	3.1	1.7	4.0	2.3	4.4	8.8
C_14:0_	4.2	2.2	5.6	6.0	5.4	3.8
C_16:0_	**26.9**	**28.4**	**28.7**	**41.4**	**31.5**	**16.6**
C_17:0_	4.2	1.3	1.6	–	tr	2.2
C_18:0_	1.7	1.3	tr	1.0	tr	1.1
C_19:0_	tr	–	1.4	2.2	1.2	–
Unsaturated:						
C_14:1_ *ω*5*c*	tr	1.1	tr	tr	tr	7.1
C_15:1_ *ω*5*c*	–	3.9	2.7	–	–	6.3
C_15:1_ *ω*6*c*	tr	1.1	1.1	tr	2.1	2.1
Cyclo-C_17:0_	**16.5**	**10.7**	**12.2**	**19.5**	**14.9**	**11.2**
Branched:						
iso-C_16:0_	–	–	2.3	–	tr	–
iso-C_17:0_	1.4	1.1	tr	tr	tr	tr
Hydroxy:						
C_10:0_ 3-OH	4.5	9.4	6.9	7.1	5.1	4.3
C_12:0_ 2-OH	–	–	–	1.9	2.3	–
Summed feature*:						
3	**20.3**	**22.4**	**18.3**	**11.9**	**19.8**	**21.4**
5	1.1	1.2	2.4	3.5	1.9	1.4
8	**13.2**	8.9	9.3	tr	8.6	8.3

Taxa: 1, strain H242^T^; 2, strain B156^T^; 3, *R. monticola* KACC 19175T; 4, *R. ginsenosidimutans* KACC 17527^T^; 5, *R. alkalitolerans* KACC 19305^T^; 6, *R. tataouinensis* KACC 11924^T^. Fatty acids amounting to less than 1.0% in all strains are not shown. Major fatty acids (> 10.0%) are highlighted in bold; tr, trace amount (< 1.0%); –, not detected. All data were obtained in this study.

*Summed features are fatty acids that cannot be resolved reliably from another fatty acid using the chromatographic conditions chosen. The MIDI system groups these fatty acids together as one feature with a single percentage of the total. Summed features 3, 5, and 8 contain C_16:1_ *ω7c* and/or C_16:1_ *ω6c*, C_18:0_ ante and/or C_18:2_ ω6,9c, and C_18:1_ *ω7c* and/or C_18:1_ *ω6c*, respectively.
