# Supplementary material for: Ramlibacter terrae sp. nov. and Ramlibacter montanisoli sp. nov., Isolated from Soil
Source: J Microbiol Biotechnol. 2021 Jul 21;31(9):1210–7. doi: 10.4014/jmb.2105.05023 (PMC9705899; doi:10.4014/jmb.2105.05023)

J. Microbiol. Biotechnol.
https://doi.org/10.4014/jmb.2105.05023

Ramlibacter terrae sp. nov. and *Ramlibacter montanisoli* sp. nov., isolated from soil

Shehzad Abid Khan†, Hyung Min Kim†, Ju Hye Baek, Hye Su Jung and Che Ok Jeon*

Department of Life Science, Chung-Ang University, Seoul 06974, Republic of Korea

†These authors have contributed equally to this work.

*Author for correspondence: Che Ok Jeon (cojeon@cau.ac.kr)

Supplementary Fig. S1. Maximum likelihood (A) and maximum parsimony (B) trees showing the phylogenetic relationships between strains H242^{T} and B156^{T} and closely related species, based on 16S rRNA gene sequences. Bootstrap values (based on 1,000 replication) greater than 70% are shown at branch points. *Neisseria gonorrhoeae* ATCC 19424^{T} (AJ247238) was used as an outgroup. The scale bars in panels A and B indicate substitutions per nucleotide position and numbers of nucleotide substitutions over the whole sequence, respectively.

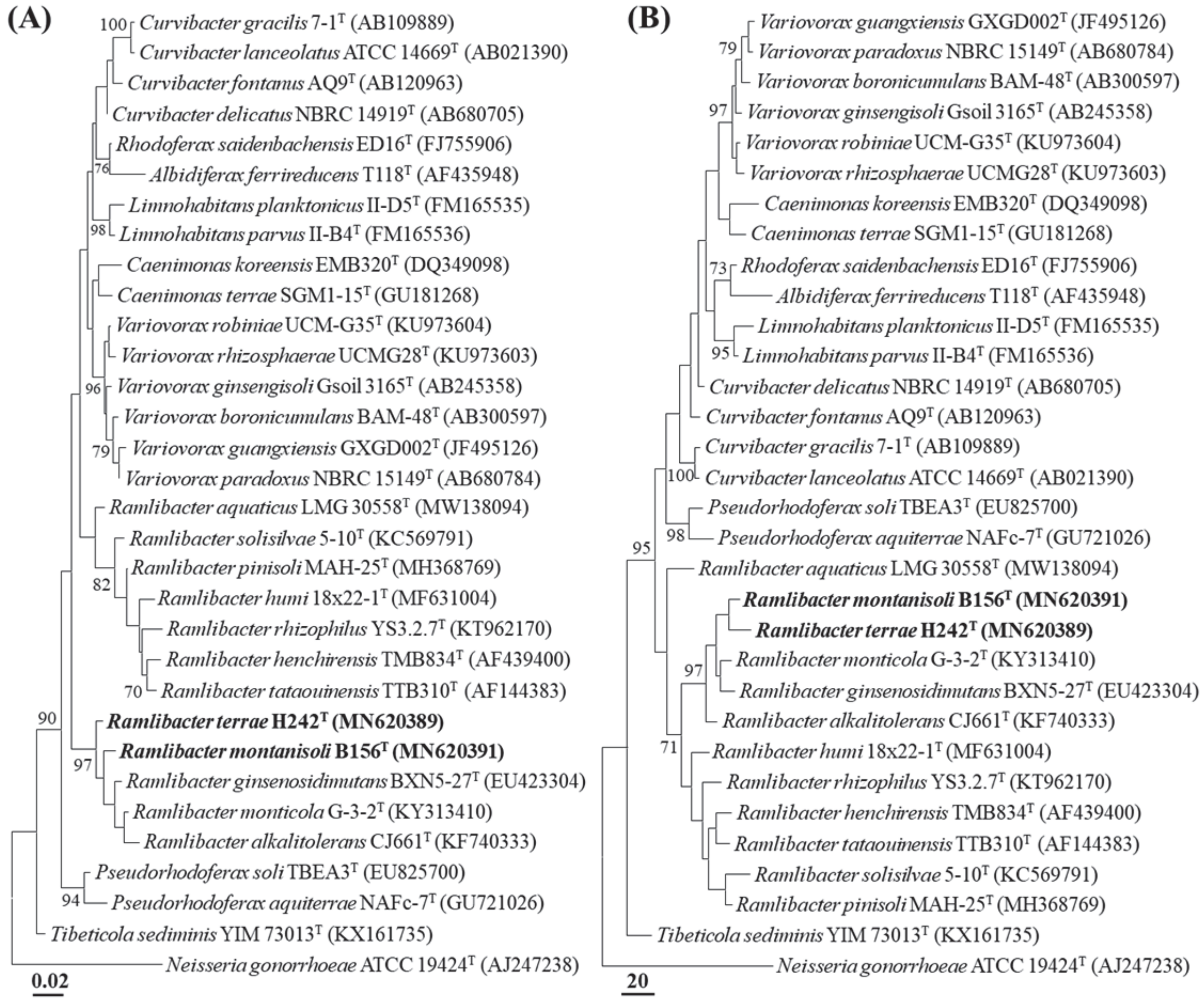

Supplementary Fig. S2. Transmission electron micrographs showing the general morphologies of negatively stained cells of strains H242^T (A) and B156^T (B) grown on R2A agar at 30 °C for 2 days. Scale bars, 0.2 μm.

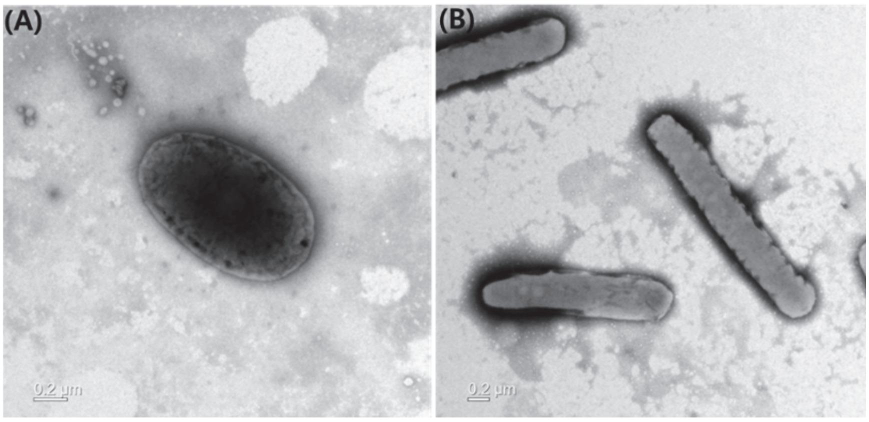

Supplementary Fig. S3. Two-dimensional thin-layer chromatograms showing the polar lipids of strains H242^T and B156^T. Solvent systems: (I) chloroform-methanol-water (65:25:4, v/v/v); (II) chloroform-acetic acid-methanol- water (80:15:12:4, v/v/v/v). The TLC plates were sprayed with 10 % ethanolic molybdophosphoric acid (A), ninhydrin (B), Dittmer-Lester reagents (C) and α-naphthol/sulfuric acid (D) for the detection of total polar lipids, aminolipids, phospholipids, and glycolipids, respectively. PG, phosphatidylglycerol; DPG, diphosphatidylglycerol; PE, phosphatidylethanolamine; APL, unidentified aminophospholipid.

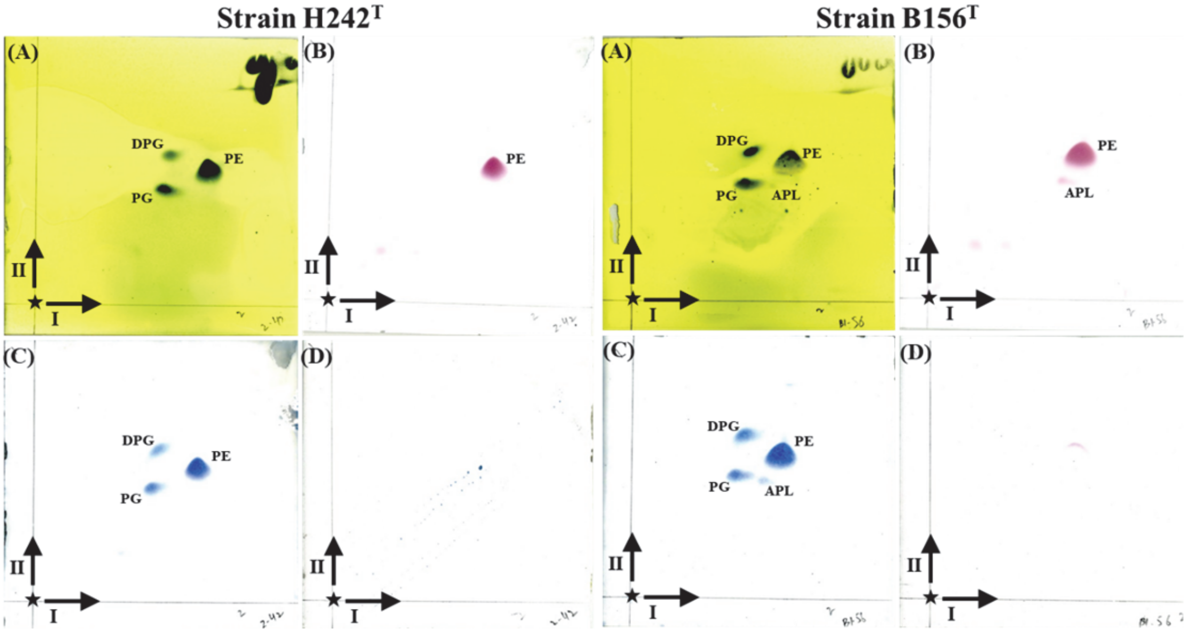

Supplement: Supplementary file 1 [file jmb-31-9-1210-supple.pdf]
